# Prognostic value of B-type natriuretic peptide (BNP) and its potential role in guiding fluid therapy in critically ill septic patients

**DOI:** 10.1186/1757-7241-20-86

**Published:** 2012-12-31

**Authors:** Zhongheng Zhang, Zhengguang Zhang, Yadong Xue, Xiao Xu, Hongying Ni

**Affiliations:** 1 Department of critical care medicine, Jinhua municipal central hospital, 351#, Mingyue Road, Jinhua, Zhejiang province, 321000, People's Republic of China; 2 Zhujiang hospital, Southern Medical University, Guangzhou, People's Republic of China; 3 Department of science and education, Jinhua municipal central hospital, Zhejiang, People's Republic of China

**Keywords:** B-type natriuretic peptide, Fluid loading, Sepsis, Outcome

## Abstract

**Background and objectives:**

The prognostic role of B-type natriuretic peptide (BNP) in septic patients is controversial. The study aimed to investigate the prognostic value of BNP in critically ill septic patients. Furthermore, because BNP is primarily released from ventricles in response to myocardial stretch, the second aim of the study was to test whether the change in BNP was correlated to the amount of fluid balance.

**Methods:**

This was a prospective observational study conducted in a tertiary 18-bed ICU. Patients fulfilled criteria of sepsis were enrolled. Exclusion criteria included renal dysfunction on ICU entry, age < 18 or >80 years old. BNP was measured on entry (BNP0) and day 3 (BNP1) and daily fluid balance over the three days were recorded. Diagnostic performances of BNP0 and ΔBNP (BNP1-BNP0) were analyzed. The correlation between fluid balance and ΔBNP were tested using Spearman’s correlation test.

**Results:**

A total of 67 subjects were eligible for the study during study period. BNP0 was significantly higher in non-survivors than in survivors (738 vs 550 pg/ml, p < 0.01). The area under curves (AUCs) of BNP0 in predicting mortality, duration of mechanical ventilation (MV) > 7 d, length of stay in ICU (LOS_ICU_) > 7 d and hospital (LOS_hospital_) > 12 d were 0.71, 0.79, 0.66 and 0.71, respectively. The AUCs of ΔBNP in predicting duration of MV > 7 d, LOS_ICU_ > 7 d and LOS_hospital_ > 12 d were 0.80, 0.84 and 0.85, respectively. The amount of fluid balance was correlated to ΔBNP (Spearman’s rho = 0.63, p < 0.01), and the correlation remained statistically significant in multivariate model.

**Conclusions:**

BNP measured on ICU entry is associated with mortality, duration of MV, LOS_ICU_ and LOS_hospital_. ΔBNP is able to predict the LOS_ICU_ and LOS_hospital_ with satisfactory sensitivity and specificity. ΔBNP is closely correlated to the amount of fluid balance during resuscitation period. However, this could only be considered as a hypothesis-generating pilot study due to its small sample size and the observational nature.

## Introduction

Sepsis is a leading cause of mortality and morbidity in intensive care unit (ICU). It is estimated that 3 in 1000 people will be affected by sepsis, and 51% of them requires ICU admission [1]. The presence of cardiovascular dysfunction in septic patients is found to be associated with worse clinical outcome [2,3]. Underlying mechanisms of cardiovascular depression include over-production of reactive oxygen species, lipid peroxidation, and impaired calcium homeostasis [4,5]. In this regard, biomarkers of cardiac dysfunction may help to identify patients at risk of death early. For instance, the conventional biomarkers of Troponin T and I have been extensively investigated in septic patients and those with elevated troponin levels were 2–3 times more likely to die [6,7].

B-type natriuretic peptide (BNP) is a 32 amino acid that is synthesized and secreted by myocytes and fibroblasts in the atria and ventricle in response to wall stress [8,9]. It has been widely accepted that BNP is a useful tool in the management of heart failure, such as diagnosis, risk stratification, and BNP-guided treatment [10-12]. More recently, many investigators have identified its value in critical care settings. Some found that BNP might be helpful in guiding fluid therapy [13], and others found that BNP was associated with clinical outcomes [14,15]. However, these results are conflicting due to the heterogeneity of study subjects. We hypothesized that 1) baseline BNP concentration was associated with clinical outcome in septic patients in ICU; and 2) change in BNP after fluid therapy was associated with clinical outcome, and the change was correlated to the amount of fluid balance.

## Materials and methods

### Study population

The study was conducted in a tertiary 18-bed ICU from September 2010 to March 2012. This was a mixed ICU enrolling both surgical and medical patients. During the study period, patients entered ICU were prospectively assessed for potential eligibility. Patients were eligible if they fulfilled the following criteria: 1) sepsis defined as systemic inflammatory response syndrome (SIRS) plus infection. SIRS was defined by the presence of more than one of the following criteria: body temperature, >38°C or <36°C; heart rate, >90 min^-1^; hyperventilation evidenced by a respiratory rate of >20 min^-1^ or a PaCO_2_ of <32 mm Hg (4.3 kPa); and a white blood cell count of >12,000 cells μL^-1^ or <4,000 μL^-1^. Infection was considered by the presence of clinical signs and inflammatory markers, and the presence of polymorphonuclear cells in normally sterile tissue or fluid and/or culture or gram stain showing a pathogenic microorganism and/or radiological evidence of an infective focus [16]. 2) patients aged between 18 and 80 years old. 3) Patients who required hemodynamic monitoring or quantitative assessment of pulmonary edema with PiCCO system. Criteria for PiCCO monitoring included hemodynamic instability, acute respiratory distress syndrome or both. Exclusion criteria were: 1) patients with acute renal injury at a degree of AKIN-2 or above [17]; 2) patients who were moribund or signed Do-not-resuscitation order (DNR); 3) patients with preexisting renal dysfunction. The study was approved by the ethic committee of Jinhua municipal central hospital. Informed consent was obtained from patient or next-of-kin.

### Data collection

On enrollment, baseline characteristics of patients were recorded, including age, sex, severity of illness as represented by Acute Physiology and Chronic Health Evaluation (APACHE II), admission diagnosis, sources of infection, hemodynamic variables (obtained by thermodilution technique). During follow up, the volumes of fluid balance in the first three days were recorded. The total net fluid balance referred to net fluid balance over the three days. Fluid balance was defined as total fluid intake including tube feeding, intravenous infusion and eating, minus total fluid output including urine output, drainage of fluid, fluid removal with renal replacement therapy and perspiration. B-type natriuretic peptide (BNP) was measured on admission (BNP0), and on the third day (BNP1). ΔBNP was calculated as BNP1 minus BNP0. BNP was measured using Triage BNP detector (Triage BNP, Biosite Company, San Diego, Calif).

The primary end point was in hospital mortality. Secondary end points included ICU length of stay (LOS_ICU_), LOS in hospital (LOS_hospital_) and duration of mechanical ventilation (MV). For patients who had been transferred to general ward and then were re-admitted to ICU within three days, days spent in general ward were considered in ICU, and LOS_ICU_ included the first and second LOS_ICU_. Mechanical ventilation included invasive mechanical ventilation and non-invasive mechanical ventilation with positive end expiratory pressure >5 cmH_2_O. In our institution, we considered tracheostomy for patients who could not wean from mechanical ventilation after day 7, thus, we used 7 days as a threshold to convert the continuous variable of duration of MV into a binary variable.

### Statistical analysis

Data were tested for normality, and expressed as mean ± SD (standard deviation) or median and interquartile range as appropriate. If data were normally distributed, the comparisons were performed using student *t* test, otherwise, Wilcoxon rank-sum (Mann–Whitney) test was used. Multivariate analysis (backward stepwise logistic regression) was performed to screen independent variables associated with mortality. Variables entered into the model were defined a priori, including age, APACHE II score, BNP0, cardiac index, and extravascular lung water; also, variables with a p < 0.1 in univariate analysis were entered into the model. The goodness-of-fit was tested using Homser-Lemeshow method. Diagnostic performance of BNP0 and ΔBNP in predicting clinical outcomes (hospital mortality, LOS_ICU_ and LOS_hospital_, duration of mechanical ventilation) were evaluated using receiver operating characteristic (ROC) curves. Diagnostic statistics including sensitivity, specificity, positive likelihood ratio (LR+), and negative likelihood ratio (LR-) were reported. The correlation between total fluid balance and ΔBNP was analyzed by using spearman’s rank correlation test. P < 0.05 with two tailed test was considered to be statistically significant. All statistical analyses were performed using the software Stata 11.0 (College Station, Texas 77845 USA).

## Results

### Patient enrollment and baseline characteristics

**Figure 1:**
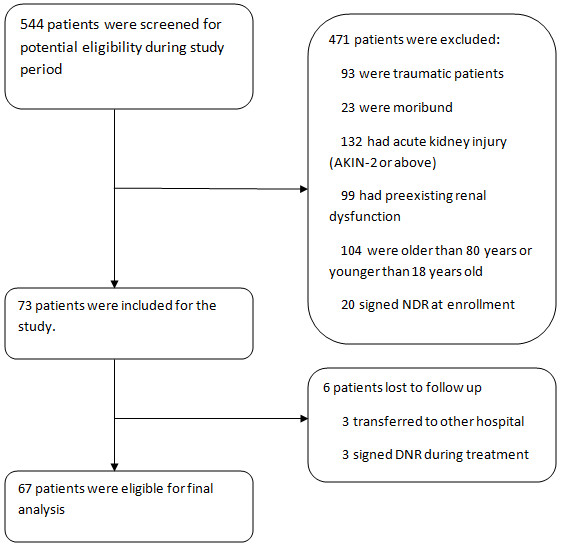
Flow chart of patients selection.

During the study period, a total of 544 patients were admitted to our ICU. 471 of them were excluded on entry due to various reasons (Figure 1), remaining 73 patients who fulfilled our inclusion criteria. During follow up, six patients were lost because three were transferred to other hospitals and the other three signed do-not-resuscitation order in the course of treatment. Finally, 67 patients were used in the analysis.

Baseline characteristics of the patients are shown in Table 1, and variables were compared between survivors and non-survivors. Survivors and non-survivors were comparable in variables including age, sex, sources of infection, proportion of patients with MV, and hemodynamic variables. Survivors had significantly lower APACHEII score than non-survivors (19 vs 31, p < 0.01). More patients in non-survivors required at least one vasopressor (66.7% vs 32.5%, p < 0.01). Fluid overload during the first two days consistently showed negative impact on survival. In total, survivors were given less fluid than non-survivors during the first three days (2051 vs 3086 ml, p = 0.03). Survivors had lower BNP levels both on entry and on day 3 than non-survivors (550 vs 738 pg/ml, p < 0.01; 594 vs 834 pg/ml, p < 0.01; respectively).

**Table 1:** Characteristics of included patients

**Variables**	**Total (n = 67)**	**Alive (n = 40)**	**Dead (n = 27)**	**P value**
Age (years)	59 ± 16	56 ± 15	64 ± 18	0.07
Sex (male, %)	43 (64.2)	25(62.5)	18 (66.7)	0.73
APACHEII	23 (19,31)	19 (16,23)	31 (25,38)	<0.01
Sources of infection (No. %)				0.47
Lung	27 (40.3)	14 (35)	13 (48.2)	
Abdomen	10 (14.9)	5 (12.5)	5 (18.5)	
Urinary tract	15 (22.4)	11 (27.5)	4 (14.8)	
Blood stream	15 (22.4)	10 (25)	5 (18.5)	
Mechanical ventilation (n, %)	31 (46.3)	16 (40)	15 (55.6)	0.21
Vasopressor use (n, %)	31 (46.3)	13 (32.5)	18 (66.7)	<0.01
Hemodynamic variables				
CVP (mmHg)	8 (6,13)	8 (6,13.5)	9 (6,12)	0.75
CI (ml/m2)	3.5 ± 1.2	3.6 ± 1.1	3.3 ± 1.4	0.30
SBP(mmHg)	130 ± 26	127 ± 24	135 ± 28	0.20
DBP(mmHg)	66 ± 15	66 ± 16	65 ± 14	0.93
HR (/min)	101 ± 21	104 ± 20	97 ± 23	0.23
SI (ml/m^2^)	35 ± 12	35 ± 11	34 ± 14	0.76
SVRI	1967 (1518,2573)	1850 (1466,2347)	2103 (1566,2917)	0.09
EVLWI	11.0 ± 7.3	10.5 ± 6.6	11.8 ± 8.2	0.45
GEDVI	746 (616,833)	744 (613,877)	751 (619,818)	0.55
Fluid balance (ml)				
Day 1	1432 (867,2133)	1003 (553,1827)	1890 (1432,2398)	<0.01
Day 2	988 (210,1322)	499 (142,1003)	1234 (994,1332)	<0.01
Day 3	324 (−324, 921)	391 (−183,1002)	122 (−1044,589)	0.16
Total	2444 (1132,3652)	2051 (874,3201)	3086 (1643,4511)	0.03
BNP0 (pg/ml)	668 (457,818)	550 (331,768)	738 (596,937)	<0.01
BNP1 (pg/ml)	689 (534,889)	594 (446,732)	834 (701,1154)	<0.01

BNP0 represents the B-type natriuretic peptide measured on ICU entry; BNP1 represents the BNP measured on day 3;

Abbreviations: *APACHEII*,Acute Physiology and Chronic Health Evaluation; *CVP*, central venous pressure; *CI*, cardiac index; *SBP*, systolic blood pressure; *DBP*, diastolic blood pressure; *HR*, heart rate; *SI*, stroke volume; *SVRI*, systemic vascular resistance index; *EVLWI*, extravascular lung water; *GEDVI*, global end diastolic volume index; *BNP*, B-type natriuretic peptide.

### BNP and clinical outcomes

In multivariate analysis (Table 2), BNP0 was found to be independently associated with in hospital survival. With each 100 pg/ml increase in BNP0, the mortality rate was doubled (OR: 2.14, 95% CI: 1.07-4.24). APACHEII score was also an independent predictor of in-hospital mortality. Variables such as vasopressor use, sex, day 1 fluid balance were excluded from the regression model by backward stepwise method. Other factors such as age and extravascular lung water were not found to be independently associated mortality. P value for the Homser-Lemeshow *χ*^2^ was 0.75, suggesting a well fitted model.

**Table 2:** Multivariate Logistic regression analysis of variables associated with mortality

**Variables**	**Odds ratio**	**95% confidence interval**	**P value**
BNP0 (with each 100 pg/ml increase)	2.14	1.07-4.27	0.03
Age	1.05	0.97-1.14	0.20
APACHEII	1.97	1.22-3.19	0.006
Cardiac output	0.25	0.06-1.01	0.05
Day 2 fluid balance (with each 100 ml increase)	1.50	1.10-2.04	0.01
Extravascular lung water	0.83	0.66-1.05	0.13

BNP0 represents the B-type natriuretic peptide measured on ICU entry.

Abbreviations: *APACHEII*, Acute Physiology and Chronic Health Evaluation; *CVP*, central venous pressure.

Diagnostic performances of BNP0 and ΔBNP in predicting clinical outcomes are shown in Table 3. BNP0 was of diagnostic value in predicting clinical outcomes, though the performance was only moderate. BNP0 was able to predict mortality with an area under receiver operating characteristic curve (AU-ROC) of 0.71. At the cutoff of 816 pg/ml, the sensitivity and specificity were 48.2% and 87.5%, respectively. The AU-ROC of BNP0 in predicting duration of MV > 7 days was 0.79, and at the cutoff of 929 pg/ml, the sensitivity and specificity were 57.1% and 88.2%, respectively. The AU-ROC of BNP0 in predicting LOS_ICU_ >7 days was 0.66, and at the cutoff of 828 pg/ml, the sensitivity and specificity were 40% and 89.2%, respectively. The AU-ROC of BNP0 in predicting hospital stay > 12 days was 0.71, and at the cutoff of 709 pg/ml, the sensitivity and specificity were 60.6% and 70.6%, respectively. ΔBNP had no value in discriminating survivors and non-survivors. However, ΔBNP was good at predicting other clinical outcomes. The AU-ROC of ΔBNP in predicting duration of MV > 7 days was 0.80, and at the cutoff of 108 pg/ml, the sensitivity and specificity were 52.9% and 85.7%, respectively. The AU-ROC of ΔBNP in predicting LOS_ICU_ > 7 days was 0.84, and at the cutoff of 101 pg/ml, the sensitivity and specificity were 73% and 80%, respectively. The AU-ROC of ΔBNP in predicting LOS_hospital_ >12 days was 0.85, and at the cutoff of 114 pg/ml, the sensitivity and specificity were 73.5% and 87.9%, respectively.

**Table 3:** Diagnostic performance of BNP0 and ΔBNP on clinical outcomes

**Outcomes**	**AU-ROC**	**Cutoff (pg/ml)**	**Sensitivity**	**Specificity**	**LR+**	**LR−**
BNP0						
Mortality	0.71 (0.59-0.84)	>816	48.2	87.5	3.85	0.59
Duration of MV > 7 days	0.79 (0.62-0.85)	>929	57.1	88.2	4.86	0.49
LOS in ICU > 7 days	0.66 (0.53-0.79)	>828	40	89.2	3.70	0.67
LOS in hospital > 12 days	0.71 (0.58-0.83)	>709	60.6	70.6	2.06	0.56
ΔBNP						
Mortality	0.58 (0.44-0.72)	>294	40.7	77.5	1.81	0.76
Duration of MV > 7 days	0.80 (0.60-0.99)	>108	52.9	85.7	3.71	0.55
LOS in ICU > 7 days	0.84 (0.74-0.93)	>101	73.0	80.0	3.65	0.34
LOS in hospital > 12 days	0.85 (0.75-0.94)	>114	73.5	87.9	6.07	0.30

BNP0 represents the B-type natriuretic peptide measured on ICU entry; ΔBNP represents the difference between BNP measured on the third day and BNP0 (BNP1-BNP0);

Abbreviation: *LOS*, length of stay; *MV*, mechanical ventilation.

### Correlation between ΔBNP and fluid balance

**Figure 2:**
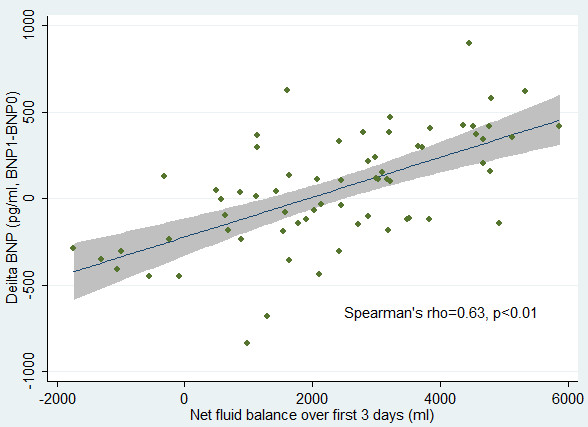
**The correlation between fluid balance and changes in B-type natriuretic peptide (ΔBNP).** ΔBNP was significantly correlated to the amount of fluid balance over the first three days (Spearman’s rho = 0.63, p < 0.01).

As shown in Figure 2, ΔBNP was significantly correlated to the fluid balance with a Spearman’s rho of 0.63 (p < 0.01). Multivariate linear regression was performed by incorporating variables that might have potential impact on BNP concentrations. These variables included age, sex, baseline BNP (BNP0), CVP, CI, and vasopressor use. The result showed that fluid balance was independently associated with ΔBNP after adjustment (coefficient: 0.1, 95% CI: 0.07-0.13; p < 0.01). With each 100 ml increase in fluid balance, the BNP increased by 10 pg/ml.

## Discussion

The study showed that elevated baseline BNP was associated with significantly increased risk of mortality, longer LOS_ICU_ and LOS_hospital_, and longer duration of MV in patients with sepsis. Furthermore, ΔBNP was also associated with poor clinical outcomes including longer duration of MV, LOS_ICU_ and LOS_hospital_. Of note, the AU-ROC of ΔBNP in predicting LOS_ICU_ and LOS_hospital_ were 0.84 and 0.85, respectively, allowing for accurate prediction of these outcomes by serial measurements of BNP. However, its diagnostic value in predicting duration of MV is suboptimal with a sensitivity and specificity of 52.9% and 85.7%, respectively. With correlation analysis, we found that ΔBNP was independently associated with the amount of net fluid balance, indicating that copious fluid loading during resuscitation period may impair cardiac function, and consequently resulting in poor clinical outcomes.

The association of elevated baseline BNP with increased risk of mortality has been proven in multiple studies [18,19]. This association may be attributable to sepsis-related cardiac depression that is characterized by myocardial stiffness and mechanical insufficiency. In response to myocardial stretch, the plasma BNP level will increase. In the present study, survivors tended to have higher cardiac index at a lower GEDVI than non-survivors, suggesting that the depressed myocardium in non-survivors required higher preload to compensate for compromised cardiac output. Our result is in line with a recent study demonstrating that elevated BNP was associated poor cardiac prognosis, and this was mediated by cardiac rebound stretch [20]. However, our result is limited by that echocardiography, which could provide more data on cardiac performance, was not obtained. This is because our department is not equipped with echocardiography. The best cutoff values used to discriminate survivors and non-survivors varied across studies, ranging from 49 to 680 pg/ml. This could be explained by the lack of assay standardization [21]. Although the currently existing BNP assays correlate closely, the lack of standardization will prohibit the communication among studies from different institutions. In our study, the cutoff value was 810 pg/ml, which was higher than that reported in the literature. This was probably due to the fact that these studies were conducted in emergency department, and the severity of illness of the study population was less than that in our study. Several studies failed to demonstrate the association of BNP with outcomes. Sturgess DJ et al. [22] showed that BNP levels were not significantly different between survivors and non-survivors (p = 0.14). However, this study enrolled only 21 subjects, lacking the statistical power to detect a difference. Furthermore, the renal function, a well known factor responsible for plasma BNP level, was not taken into consideration in their study. The sensitivity of BNP in predicting outcomes is low, that is, normal BNP will not guarantee a good clinical outcome in critically ill patients. This supports the notion that there is no single biomarker that can be used for clinical decision making in critically ill patients and comprehensive clinical assessment is indispensible.

The identification of baseline BNP as a predictor of outcome is not the end of investigation. The next question would focus on whether the changes in BNP during ICU stay could influence the outcome, and whether modifiable factors that are responsible for the change in BNP could be identified. The present study showed that ΔBNP performed well in predicting LOS_ICU_ and LOS_hospital_, as well as the duration of MV. To the best of our knowledge, our study is the first to describe this association. Furthermore, we investigated factors that would influence the changes in BNP. In multivariate regression model, we found that the amount of fluid balance was independently associated with ΔBNP. This suggests that fluid overloading is responsible for the increase in BNP during treatment, and copious fluid infusion appears to have negative impact on clinical outcome. BNP can be used as an indicator to tell whether fluid loading is too much. If BNP increases by more than 100 pg/ml during resuscitation, fluid should be withheld and conservative strategy could be performed. When patients are hemodynamically stable, negative fluid balance may be beneficial. Actually, the negative impact of fluid overloading in critically ill patients is not new. In a landmark study, Wiedemann HP and coworkers demonstrated the adverse effect of fluid overloading in patients with ARDS, and recommended the conservative fluid strategy in this group of patients [23]. Similar results have been replicated in another study [24]. In septic patients, although the early goal directed therapy (EGDT) requires copious fluid infusion during the initial six hours, a growing body of evidence favors the conservative strategy of fluid management [25-27]. However, although it seems clear that less fluid is better, the quantification of the conservative strategy remains to be addressed. In our study, we showed that an increase in BNP > 100 pg/ml could predict extended LOS in ICU and hospital, and the longer duration of MV. The increase in BNP was significantly correlated to net fluid balance. However, the correlation coefficient is low, indicating that there are many other factors that are responsible for plasma BNP concentration. These well known factors responsible for BNP concentration include sex, renal function, age, and tachycardia [28,29]. From a clinical perspective, BNP alone cannot replace hemodynamic monitoring for fluid resuscitation.

There are several limitations in our study. First, the study is observational in nature and the conclusions can only be served as hypothesis-generating, and this study could be considered a pilot study. Further randomized controlled trials can be performed by using BNP-directed fluid management protocol. Fluid infusion in the experimental arm will target an increase in BNP level of less than 100 pg/ml. However, before such trials can be started, other confounding factors of plasma BNP should be properly defined. Second, patients with significant renal dysfunction were excluded from the current analysis, and thus the results cannot be applied to patient with renal impairment. Because a substantial number of patients with severe sepsis present renal dysfunction, further investigations focusing on this group of patients are mandatory. Third, the study took place from ICU entry to the next three days, and it is not within the most effective “early window” of resuscitation period. Further studies conducted in the emergency department may overcome such shortcomings. Forth, this study was conducted in a single center, whether the conclusions can be generalized to other institutions requires further multi-center studies.

In aggregate, the study confirmed previous findings that elevated baseline BNP was predictive of poor clinical outcomes, and a ΔBNP > 100 pg/ml was predictive of LOS_ICU_ >7 d and LOS_hospital_ > 12 d. What’s new and provocative in our study is that ΔBNP is significantly correlated to the amount of fluid balance. However, because the correlation coefficient is low and there are many other factors that are responsible for BNP concentration, BNP alone cannot be used for the monitoring of fluid resuscitation.

## Abbreviations

BNP: B-type natriuretic peptide; AUC: Area under curve; ICU: Intensive care unit; LOS: Length of stay; AKIN: Acute kidney injury network; MV: Mechanical ventilation; APACHE II: Acute Physiology and Chronic Health Evaluation; CVP: Central venous pressure.

## Competing interests

The authors declare that they have no competing interests.

## Authors’ contributions

ZZ contributed to the coordinating, enrolling and monitoring of the study subjects. Zhengguang Z has contributed to the interpretation of data and manuscript drafting; YX has contributed to the statistical analysis and data interpretation; HN has contributed to the study protocol and is including study participants. XX is enrolling subjects and collecting data, and she is also responsible for the monitoring of safety. This study was partly supported by the Zhejiang Provincial Natural Science Foundation of China (Q12B050005). All authors read and approved the final manuscript.
